# Observation of a new type of aggregation-induced emission in nanoclusters†
†Electronic supplementary information (ESI) available: Fig. S1–S13 for the ESI-MS, UV-vis, PL, and ^31^P NMR spectra and the structural anatomies of the NCs. See DOI: 10.1039/c7sc05317g


**DOI:** 10.1039/c7sc05317g

**Published:** 2018-02-19

**Authors:** Xi Kang, Shuxin Wang, Manzhou Zhu

**Affiliations:** a Department of Chemistry , Center for Atomic Engineering of Advanced Materials , Anhui Province Key Laboratory of Chemistry for Inorganic/Organic Hybrid Functionalized Materials , Anhui University , Hefei , Anhui 230601 , China . Email: ixing@ahu.edu.cn ; Email: zmz@ahu.edu.cn

## Abstract

The strategy of aggregation-induced emission (AIE) has been widely used to enhance the photo-luminescence (PL) in the nanocluster (NC) research field. Most of the previous reports on aggregation-induced enhancement of fluorescence in NCs are induced by the restriction of intramolecular motion (RIM). In this work, a novel mechanism involving the restriction of the “dissociation–aggregation pattern” of ligands is presented using a Ag_29_(BDT)_12_(TPP)_4_ NC (BDT: 1,3-benzenedithiol; TPP: triphenylphosphine) as a model. By the addition of TPP into an *N*,*N*-dimethylformamide solution of Ag_29_(BDT)_12_(TPP)_4_, the PL intensity of the Ag_29_(BDT)_12_(TPP)_4_ NC could be significantly enhanced (13 times, quantum yield from 0.9% to 11.7%) due to the restricted TPP dissociation–aggregation process. This novel mechanism is further validated by a low-temperature PL study. Different from the significant PL enhancement of the Ag_29_(BDT)_12_(TPP)_4_ NC, the non-dissociative Pt_1_Ag_28_(S-Adm)_18_(TPP)_4_ NC (S-Adm: 1-adamantanethiol) exhibits a maintained PL intensity under the same TPP-addition conditions. Overall, this work presents a new mechanism for largely enhancing the PL of NCs *via* modulating the dissociation of ligands on the NC surface, which is totally different from the previously reported AIE phenomena in the NC field.

## Abstract 2

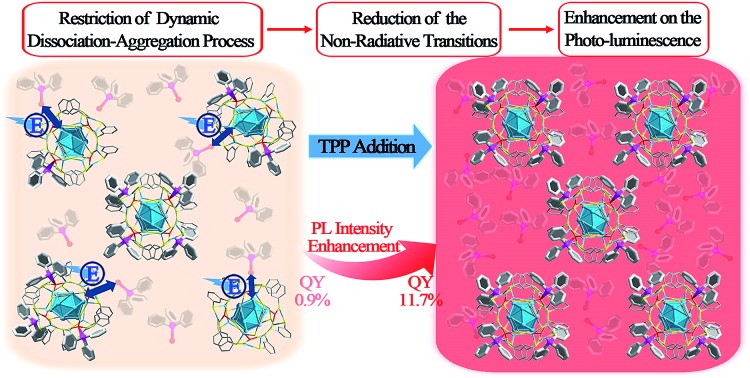
A novel mechanism of aggregation-induced emission defined as the restriction of the ligand dissociation–aggregation process in the nanocluster range is proposed.

## 1 Introduction

Noble metal nanoclusters (NCs) have attracted widespread attention due to their advantages of atomically precise structures, distinct physical/chemical properties, and extensive catalytic/biomedical applications.^1^–^16^ Among these interesting physical/chemical properties, photo-luminescence (PL) represents one of the most fascinating features owing to the low toxicity, good photostability, and high biocompatibility of NCs.^1^,^2^,^8^,^17^–^24^ Although several luminescent NCs have been reported,^8^,^18^,^21^–^31^ most of them exhibit lower quantum yields (QYs) compared with other fluorescent nanomaterials (such as lanthanide nanoparticles,^32^ organic dyes^33^ and quantum dots^34^), which severely impedes extensive application of fluorescent NCs in biological and sensing fields. Several strategies have been developed to enhance the PL QY of NCs, and they can be mainly classified into the following three categories: (1) tailoring the capping ligands of NCs (in terms of controlling the ligand to metal charge transfer (LMCT) process);^8^,^35^–^37^ (2) controlling the metal composition in the NC kernel;^21^,^22^,^26^,^28^,^29^,^38^ and (3) aggregation-induced emission (AIE) from non- or weakly luminescent metal NCs (or complexes).^19^,^20^,^25^,^39^,^40^ Nowadays, the AIE strategy is being expanded to the hydrocarbon, metal complex, metal NC, and macromolecular research fields.^41^ The corresponding AIEgens have been widely applied in biological probing, chemical sensing, and organic light-emitting diodes, to name a few.^42^ In the NC field, the AIE strategy could boost the PL QY through facile approaches (*e.g.*, solvent- or cation-inducing approaches).^19^,^25^ Furthermore, the previously reported enhancements of fluorescence in NCs have mostly been achieved by the restriction of intramolecular motion (RIM, a general mechanism in AIE materials).^19^,^25^ This raises some interesting questions: are there any other patterns (other than RIM) of AIE that exist in the NC field? If so, how can we enhance the QY of fluorescent NCs with new patterns? Addressing these issues will not only develop a powerful and practical strategy for synthesizing more fluorescent NCs with enhanced PL QYs, but also promote wide-range application of AIE in the NC field.

**Scheme 1:**
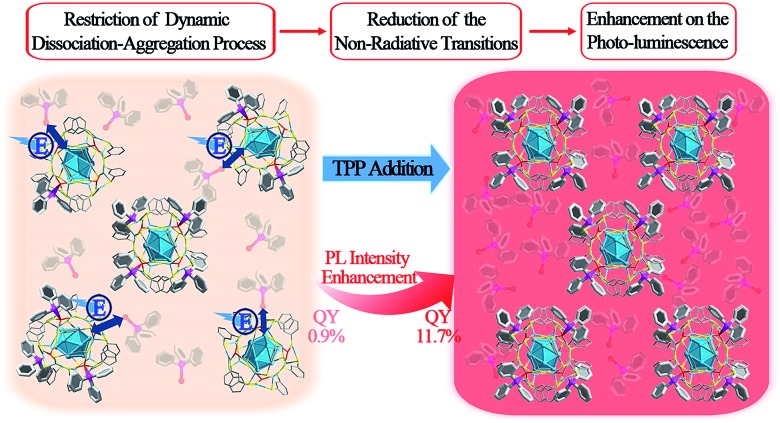
Enhancement of PL intensity induced by the restriction of the TPP dissociation–aggregation process based on the Ag_29_(BDT)_12_(TPP)_4_ NC in the solution state.

Herein, a novel AIE pattern (the restriction of the ligand dissociation–aggregation process) is discovered using a Ag_29_(BDT)_12_(TPP)_4_ NC (BDT, 1,3-benzenedithiol; TPP, triphenylphosphine) as a model. The PL intensity of the Ag_29_(BDT)_12_(TPP)_4_ NC in the solid or crystal state is significantly higher than that in the solution state. Considering the particularly close packing of Ag_29_(BDT)_12_(TPP)_4_ as well as the existence of π···π and C–H···π interactions between the ligands, the AIE process is unlikely caused by the RIM completely in the case of the Ag_29_(BDT)_12_(TPP)_4_ NC. Furthermore, electrospray ionization mass spectrometry (ESI-MS) and ^31^P nuclear magnetic resonance (^31^P NMR) analyses are performed on the Ag_29_(BDT)_12_(TPP)_4_ NC, and they reveal several dissociated ligands from Ag_29_(BDT)_12_(TPP)_4_; that is, the mechanism of AIE in Ag_29_(BDT)_12_(TPP)_4_ could be the restriction of the TPP dissociation–aggregation process. To prove the mechanism, different concentrations of TPP molecules are added to the dissociative Ag_29_(BDT)_12_(TPP)_4_ and non-dissociative Pt_1_Ag_28_(S-Adm)_18_(TPP)_4_ (S-Adm, 1-adamantanethiol) NC solution. Resultantly, the PL intensity of Ag_29_(BDT)_12_(TPP)_4_ is significantly enhanced (up to 13 times, with a PL QY from 0.9% to 11.7%) by the addition of TPP owing to the restriction of the TPP dissociation–aggregation process (or the chemical equilibrium shifting to TPP aggregation on the Ag_29_(BDT)_12_(TPP)_*x*_ NCs, summarized in Scheme 1). By contrast, the non-dissociative Pt_1_Ag_28_(S-Adm)_18_(TPP)_4_ shows nearly the same intensity of PL after the addition of TPP. In addition, the temperature-dependent PL of the Ag_29_(BDT)_12_(TPP)_4_ NC shows two segments of rising curves, which represent both the restriction of the TPP dissociation–aggregation process (the QY varies from 0.9% to 22.5%) and the quenched thermal vibration process (the QY varies from 22.5% to about 100%). However, the non-dissociative Pt_1_Ag_28_(S-Adm)_18_(TPP)_4_ only exhibits a quenched thermal vibration process (the QY varies from 9.3% to ∼100%).

## 2 Experimental methods

### Materials

All chemicals including silver nitrate (AgNO_3_, 99%, metal basis), hexachloroplatinic(iv) acid (H_2_PtCl_6_·6H_2_O, 99.99%, metals basis), triphenylphosphine (TPP, 99%), benzene-1,3-dithiol (BDT, 99%), 2,4-dimethylbenzenethiol (HSPhMe_2_, 99%), 1-adamantanethiol (HSC_10_H_15_, 99%), sodium borohydride (NaBH_4_, 99.9%), methylene chloride (CH_2_Cl_2_, HPLC grade, Aldrich), acetic ether (CH_3_COOC_2_H_5_, HPLC, Aldrich), methanol (CH_3_OH, HPLC, Aldrich), ethanol (CH_3_CH_2_OH, HPLC, Aldrich) and *N*,*N*-dimethylformamide (DMF, HPLC, Aldrich) were purchased from Sigma-Aldrich and used without further purification. Pure water was purchased from Wahaha Co. Ltd. All glassware was thoroughly cleaned with aqua regia (HCl : HNO_3_ = 3 : 1 v/v), rinsed with copious amounts of pure water, and then dried in an oven prior to use.

### Synthesis of the Ag_29_(BDT)_12_(TPP)_4_ NC

The synthesis of the Ag_29_(BDT)_12_(TPP)_4_ NC was performed following a method reported by Bakr and coworkers.^27^

### Synthesis of the Pt_1_Ag_28_(S-Adm)_18_(TPP)_4_ NC

The synthesis of the Pt_1_Ag_28_(S-Adm)_18_(TPP)_4_ NC was performed according to our previous work.^43^

### Synthesis of the Pt_1_Ag_28_(BDT)_12_(TPP)_4_ NC

The synthesis of the Pt_1_Ag_28_(BDT)_12_(TPP)_4_ NC was performed following a method previously reported by Bakr and coworkers.^44^

### Test of the TPP ligand concentration–PL intensity correlation

10 mg of Ag_29_(BDT)_12_(TPP)_4_ (or Pt_1_Ag_28_(S-Adm)_18_(TPP)_4_ or Pt_1_Ag_28_(BDT)_12_(TPP)_4_) NC was dissolved in 10 mL of DMF. Then the TPP ligand was added to the DMF solution with the molar ratio of TPP/NC ranging from 0.1 to 10. The PL spectra were then measured for these mixed solutions.

### Test of the temperature–PL intensity correlation

10 mg of Ag_29_(BDT)_12_(TPP)_4_ (or Pt_1_Ag_28_(S-Adm)_18_(TPP)_4_) NC was dissolved in 10 mL of DMF. Then the solutions were cooled to different temperatures and the PL spectra were measured.

### Characterization

All UV-vis absorption spectra of NCs dissolved in DMF were recorded using an Agilent 8453 diode array spectrometer, with the background corrected by using a DMF blank. Solid samples were dissolved in DMF to make a dilute solution, which was transferred to a 1 cm path length quartz cuvette for spectral measurements.

PL spectra were measured on an FL-4500 spectrofluorometer with the same optical density (OD) of ∼0.05. In these experiments, the NC solutions were prepared in DMF at a concentration of less than 1 mg mL^–1^.

Absolute quantum yields (QYs) were measured with dilute solutions of NCs (0.05 OD absorption at 445 nm) on a HORIBA FluoroMax-4P.

^31^P NMR spectra were acquired using a Bruker 600 Avance III spectrometer equipped with a Bruker BBO multinuclear probe (BrukerBioSpin, Rheinstetten, Germany). To achieve a sufficient signal-to-noise ratio, the ^31^P NMR spectra were recorded by collecting 1k scans with a recycle delay time of 5 s.

Electrospray ionization time-of-flight mass spectrometry (ESI-TOF-MS) measurements were performed on a MicrOTOF-QIII high-resolution mass spectrometer.

## 3 Results and discussion

**Fig. 1:**
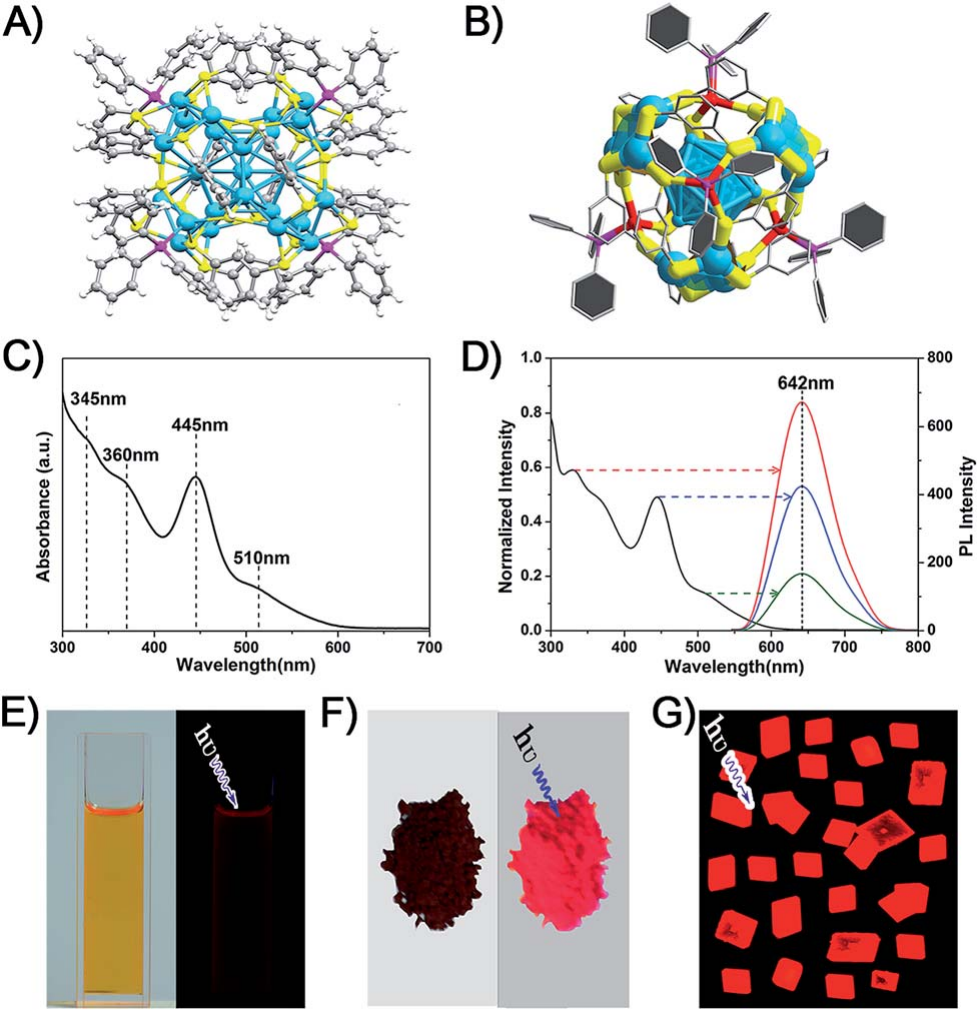
(A) Total structure of the Ag_29_(BDT)_12_(TPP)_4_ NC (redrawn from ref. 27). (B) Structural anatomy of the Ag_29_(BDT)_12_(TPP)_4_ NC. (C) UV-vis spectrum of the Ag_29_(BDT)_12_(TPP)_4_ NC (dissolved in DMF). (D) PL excitation spectrum (black) and emission spectra (other colors) of the Ag_29_(BDT)_12_(TPP)_4_ NC. Digital photographs of the Ag_29_(BDT)_12_(TPP)_4_ NC in (E) solution state, (F) solid state, and (G) crystal state under visible or UV light. Color codes: cerulean/red spheres, Ag; yellow spheres, S; purple spheres, P; grey spheres, C; white spheres, H.

The Ag_29_(BDT)_12_(TPP)_4_ NC was prepared following a method previously published by Bakr and coworkers.^27^ They also reported the crystal structure of Ag_29_(BDT)_12_(TPP)_4_.^27^ The structure is shown in Fig. 1A and B. Regarding the structural anatomy, Ag_29_(BDT)_12_(TPP)_4_ is composed of a three-layer configuration: the icosahedral Ag_13_ kernel is enclosed by four Ag_3_S_6_ motifs to form a Ag_25_(BDT)_12_ architecture (it should be noted that the four Ag_3_S_6_ motifs are united by the BDT ligands); the Ag_25_(BDT)_12_ architecture is further capped by four Ag–TPP units. Fig. 1C shows the UV-vis spectrum of Ag_29_(BDT)_12_(TPP)_4_, which exhibits broad, multiband optical absorption bands centred at 345, 360, 445, and 510 nm. Furthermore, Ag_29_(BDT)_12_(TPP)_4_ possesses an emission band centred at 642 nm (Fig. 1D). Additionally, the PL excitation spectrum is almost the same as the absorption spectrum (Fig. 1C and D), which is reminiscent of some previously reported fluorescent NCs as well as the quantum-dot behavior.^22^,^25^,^45^

It is noteworthy that the PL QY of Ag_29_(BDT)_12_(TPP)_4_ is only 0.9%, which is too weak to be perceived by the naked eye (Fig. 1E).^27^,^28^ Interestingly, the Ag_29_(BDT)_12_(TPP)_4_ NC exhibits enhanced fluorescence in the solid or crystal state (Fig. 1F and G). These observations suggest that the aggregation process significantly boosts the emission of Ag_29_(BDT)_12_(TPP)_4_. Previously, the RIM pattern was believed to be the primary cause either in the solvent- or ion-induced AIE process.^19^,^25^ In our current work, the AIE observed during the drying or crystallization process is obviously not relevant to any ionic effect, because our process only involves a solvent evaporation process.

**Fig. 2:**
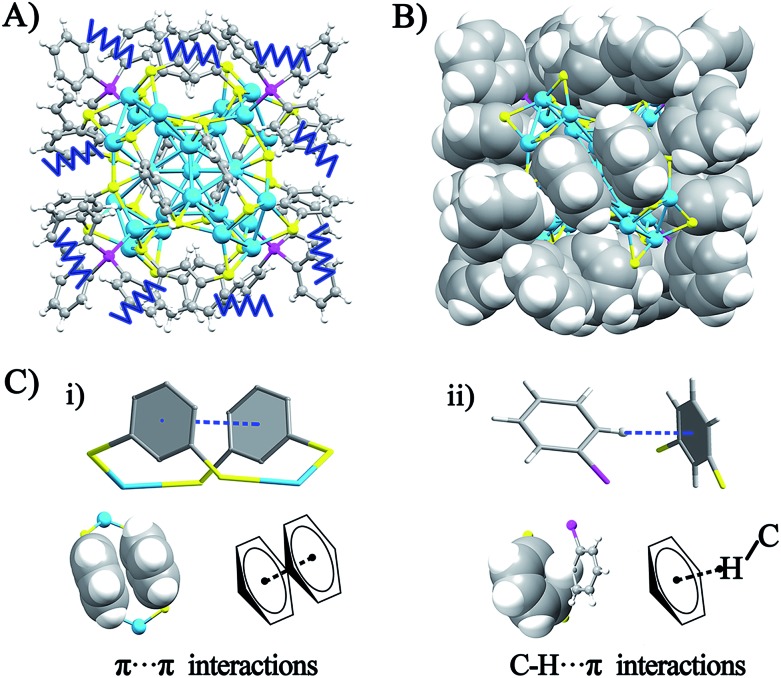
(A) Illustration of the vibration patterns in the Ag_29_(BDT)_12_(TPP)_4_ NC. (B) The space-fill pattern of the crystal structure of the Ag_29_(BDT)_12_(TPP)_4_ NC. (C) (i) The π···π interaction between neighboring benzene rings on BDT ligands, and (ii) the C–H···π interaction between *ortho*-position C–H on TPP and benzene rings on BDT ligands. Color codes: cerulean spheres, Ag; yellow spheres, S; purple spheres, P; grey spheres, C; white spheres, H.

The crystal structure of the Ag_29_(BDT)_12_(TPP)_4_ NC is analyzed in order to gain insight into the aforementioned solvent evaporation-induced AIE. In the solution state, the energy dissipation of photo-excited Ag_29_(BDT)_12_(TPP)_4_ includes two pathways: (i) non-radiative transitions (mainly affected by intra-molecular vibrations), and (ii) radiative transitions (through luminescence). Fig. 2A shows an illustration of intra-molecular vibrations of the Ag_29_(BDT)_12_(TPP)_4_ NC, which comprise rota-vibrations and swing-vibrations. However, the considerable close-packing of the structural configuration (see the space-fill pattern in Fig. 2B) suggests that such vibrations are difficult. Furthermore, as mentioned above, each Ag_3_S_6_ motif is linked by the BDT ligand, which restricts the vibrations of Ag–S–Ag motifs. Moreover, many π···π and C–H···π interactions among BDT or TPP ligands are observed. Specifically, every two neighboring benzene rings of BDT ligands interact through π···π interaction, which forms 6 pairs of π···π interactions in total (Fig. 2C). In addition, the vibration of each of the *ortho*-position C–H on TPP is rendered difficult owing to the C–H···π interaction with the nearby benzene ring of BDT (altogether 12 pairs of C–H···π interactions, shown in Fig. 2C). To summarize, the main cause of the weak PL of the Ag_29_(BDT)_12_(TPP)_4_ NC in solution is not thermal vibration, because the architecture of the Ag_29_(BDT)_12_(TPP)_4_ NC exhibits strong rigidity. Consequently, the AIE of the Ag_29_(BDT)_12_(TPP)_4_ NC is unlikely induced by the RIM pattern completely.

**Fig. 3:**
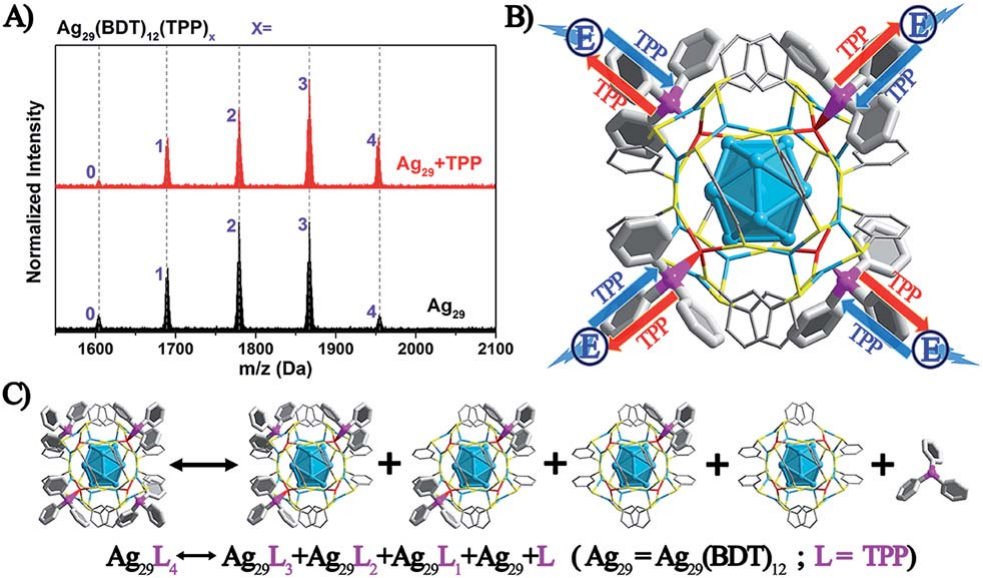
(A) ESI-MS spectra of the Ag_29_(BDT)_12_(TPP)_4_ NC with or without the addition of TPP. (B) Illustration of the dynamic TPP dissociation–aggregation process on the Ag_29_(BDT)_12_(TPP)_4_ and the corresponding energy loss. (C) The dynamic TPP dissociation–aggregation reversible reaction. Color codes: cerulean/red spheres, Ag; yellow spheres, S; purple spheres, P; grey spheres, C; white spheres, H.

The above discussion raises an interesting question: what is the essential role of the AIE in the Ag_29_(BDT)_12_(TPP)_4_ NC? From the ESI-MS spectrum of Ag_29_(BDT)_12_(TPP)_4_ (Fig. 3A, see Fig. S1† for the expansion of the spectrum, and Fig. S2† for the comparison of the experimental and simulated isotope patterns), we find that the TPP ligands are capable of dissociation to generate Ag_29_(BDT)_12_(TPP)_*x*_ (*x* = 0–3) and the dissociated TPP ligands. For instance, the highest signal in the ESI-MS spectrum is the peak of Ag_29_(BDT)_12_(TPP)_2_, which is generated by dissociating two TPP ligands of the intact NC. To further validate the TPP dissociation process of Ag_29_(BDT)_12_(TPP)_4_ in the solution state, ESI-MS measurements were also performed on the mixture of Ag_29_(BDT)_12_(TPP)_4_ NC with extra TPP. The spectrum (Fig. 3A, red line) exhibits a stronger signal of the intact Ag_29_(BDT)_12_(TPP)_4_ NC compared with the case of the Ag_29_(BDT)_12_(TPP)_4_ NC only (Fig. 3A, black line), which indicates a suppressed dissociation process with extra TPP in solution (note that because of the dilution process in the ESI-MS measurements, there were still lots of dissociated Ag_29_(BDT)_12_(TPP)_*x*_ signals in the sample mixture). Therefore, based on the ESI-MS results, the reversible reaction of the Ag_29_(BDT)_12_(TPP)_4_ NC in solution could be determined to be Ag_29_(BDT)_12_(TPP)_4_ ↔ Ag_29_(BDT)_12_(TPP)_3_ + Ag_29_(BDT)_12_(TPP)_2_ + Ag_29_(BDT)_12_(TPP)_1_ + Ag_29_(BDT)_12_ + TPP (shown in Fig. 3C). Accordingly, the TPP ligands on the Ag_29_(BDT)_12_(TPP)_4_ NC are in dynamic dissociation/aggregation. It is suggested that the breaking of coordination bonds would consume energy,^46^ and thus the energy loss with non-radiative transitions would influence the energy release with the radiative transition. Specifically, the TPP dissociation process would consume vast amounts of energy, which could be used to explain the result of low PL of the photo-excited Ag_29_(BDT)_12_(TPP)_4_ NC in the solution state. Thus, we speculate that the significantly enhanced PL of the Ag_29_(BDT)_12_(TPP)_4_ NC in the solid or crystal state should arise from the restriction of the TPP dissociation–aggregation process—which inhibits the non-radiative pathways, and thus enhances the radiative pathway (PL). Therefore, the AIE of the Ag_29_(BDT)_12_(TPP)_4_ NC could be induced by the restriction of the ligand dissociation–aggregation process.

**Fig. 4:**
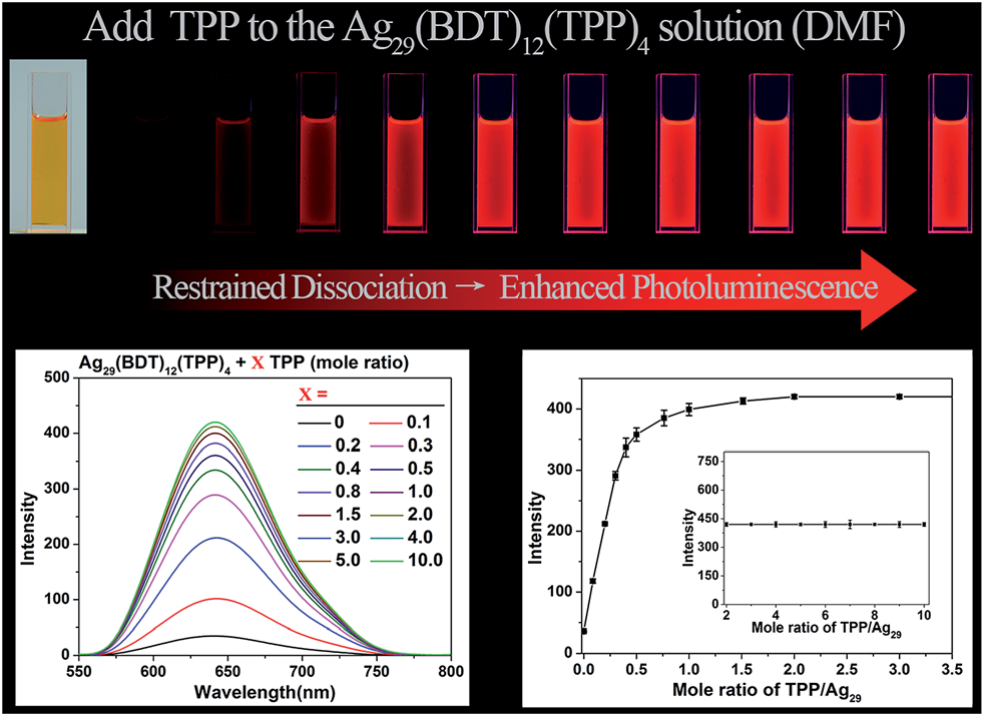
PL variation trend of the Ag_29_(BDT)_12_(TPP)_4_ NC resulting from the addition of different molar ratios of TPP ligand. (Top) PL intensity variation monitored by digital photography of the Ag_29_(BDT)_12_(TPP)_4_ NC in the solution state under UV light. (Bottom-left) PL intensity variation monitored using the fluorescence spectrometer. (Bottom-right) PL intensity variation monitored at the fixed point of 642 nm.

Considering that the TPP dissociation–aggregation process is a reversible reaction, we are motivated to control the reversible process to control the intensity of PL. As shown in Fig. 4, an increasing proportion (molar ratio) of TPP added to the DMF solution of Ag_29_(BDT)_12_(TPP)_4_ increases the PL intensity rapidly in the beginning, and then it levels off, with the highest PL QY being 11.7%, which is in striking contrast to the weakly luminescent Ag_29_(BDT)_12_(TPP)_4_ NC with no TPP addition (QY = 0.9%). A quantitative test was performed to obtain the relationship between the PL intensity and the amount of TPP added (Fig. 4, bottom-left). When adding a 0.1 molar ratio of TPP (*versus* NC) to the DMF solution of Ag_29_(BDT)_12_(TPP)_4_ NC, the PL intensity increases by almost 3 times compared with the initial state. Furthermore, by the further addition of TPP, the QY enhancement gradually becomes steady. Finally, a 13-fold enhancement compared to the initial state is achieved when the amount of added TPP is greater than 2 (molar ratio). The PL intensity at 642 nm is compared (Fig. 4, bottom-right), which also confirms the PL variation trend in the PL QY test (*i.e.*, the increasing trend as well as the 13-fold enhancement). To sum up, the redundant TPP ligands will prevent the TPP dissociation–aggregation process on the nanocluster surface, and then enhance the PL intensity of the Ag_29_(BDT)_12_(TPP)_4_ NC.

**Fig. 5:**
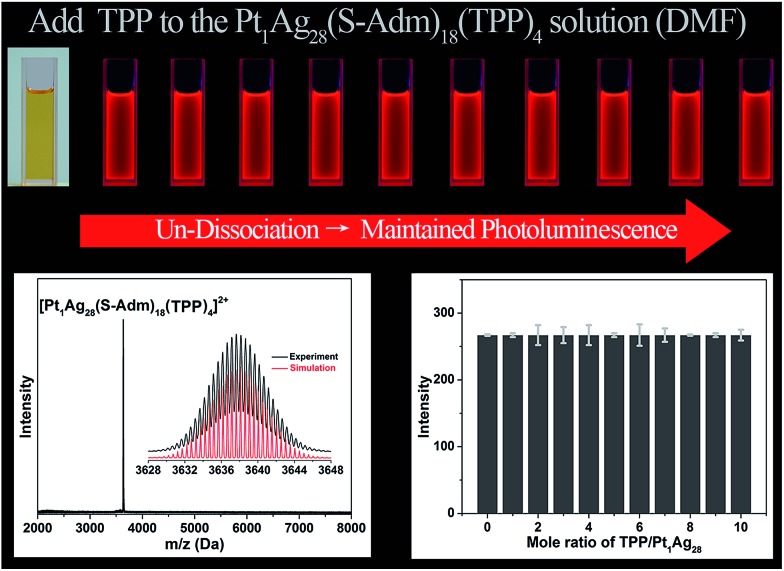
PL variation trend of the Pt_1_Ag_28_(S-Adm)_18_(TPP)_4_ NC resulting from the addition of different molar ratios of TPP. (Top) PL intensity variation monitored using the digital photographs of the Pt_1_Ag_28_(S-Adm)_18_(TPP)_4_ NC in the solution state under UV light. (Bottom-left) ESI-MS spectrum of the Pt_1_Ag_28_(S-Adm)_18_(TPP)_4_ NC. Insets: experimental and simulated isotope patterns. (Bottom-right) PL intensity variation monitored at the fixed point of 672 nm.

For comparison, the same TPP-addition experiment was performed on the Pt_1_Ag_28_(S-Adm)_18_(TPP)_4_ NC (see Fig. S3† for the structural anatomy of Pt_1_Ag_28_(S-Adm)_18_(TPP)_4_).^43^ The ESI-MS of the Pt_1_Ag_28_(S-Adm)_18_(TPP)_4_ NC shows a distinct peak at 3637.63 Da (Fig. 5, bottom-left), which clearly illustrates the non-dissociated state of TPP ligands of this NC in the DMF solution. The unchanged ESI-MS and UV-vis spectra after the addition of TPP ligands illustrate that the as-synthesized nanoclusters will not decompose or transform in this operation (Fig. S4†). Importantly, in sharp contrast to the PL variation trend of the Ag_29_(BDT)_12_(TPP)_4_ NC, the PL intensity of the Pt_1_Ag_28_(S-Adm)_18_(TPP)_4_ NC maintains a QY of 9.3% no matter how high the TPP amount added to the solution is (Fig. 5, top). Note that the PL QY of Pt_1_Ag_28_(S-Adm)_18_(TPP)_4_ in CH_2_Cl_2_ is 4.9%,^43^ which illustrates the solvent effect on the PL of Pt_1_Ag_28_(S-Adm)_18_(TPP)_4_. The PL intensity of the Pt_1_Ag_28_(S-Adm)_18_(TPP)_4_ NC monitored at 672 nm by fluorescence spectroscopy also indicates the unchanged fluorescence (Fig. 5, bottom-right). In other words, the non-dissociative Pt_1_Ag_28_(S-Adm)_18_(TPP)_4_ NC does not display any equilibrium dissociation–aggregation process, and thus the addition of TPP would not alter the PL intensity in the solution state. The sharp contrast between the PL variation trends of the Ag_29_(BDT)_12_(TPP)_4_ and Pt_1_Ag_28_(S-Adm)_18_(TPP)_4_ NCs indicates that the restriction of the ligand dissociation–aggregation process should be the major underlying mechanism of AIE in the Ag_29_(BDT)_12_(TPP)_4_ NC.

Additionally, in order to structurally and compositionally match the Pt_1_Ag_28_(S-Adm)_18_(TPP)_4_ NC, the TPP-addition experiment was performed on a Pt centrally doped Ag_29_(BDT)_12_(TPP)_4_ NC (that is, Pt_1_Ag_28_(BDT)_12_(TPP)_4_ NC).^44^ As shown in Fig. S5,† the PL intensity of Pt_1_Ag_28_(BDT)_12_(TPP)_4_ also exhibits significant enhancement by the addition of TPP (the maximum PL QY was 18.9%), which is similar to the case of Ag_29_(BDT)_12_(TPP)_4_; hence, Pt doping is not the critical factor for PL enhancement with TPP addition. Specifically, the fluorescence intensity (centered at 720 nm) rises rapidly during the initial TPP addition and then levels off once the molar ratio (TPP to Pt_1_Ag_28_(BDT)_12_(TPP)_4_ NC) is greater than 1.5. A 7-fold enhancement is finally obtained by the addition of TPP to the Pt_1_Ag_28_(BDT)_12_(TPP)_4_ NC. Considering the same PL enhancement phenomenon of the Pt_1_Ag_28_(BDT)_12_(TPP)_4_ and Ag_29_(BDT)_12_(TPP)_4_ NCs with the addition of TPP ligands, the difference in the metallic composition is unlikely to be the primary cause for being dissociative or not of these NCs. The major different between the dissociative Ag_29_(BDT)_12_(TPP)_4_ and non-dissociative Pt_1_Ag_28_(S-Adm)_18_(TPP)_4_ nanoclusters is the outer ligands (BDT *vs.* S-Adm). The difference of these two ligands largely affects the structure of the metallic kernel and outer complex shell, which is a critical factor in the dissociative/non-dissociative phenomenon (see Fig. S6† for the structural anatomies of the Pt_1_Ag_28_(BDT)_12_(TPP)_4_ as well as the Pt_1_Ag_28_(S-Adm)_18_(TPP)_4_).

^31^P NMR was performed to validate the TPP dynamic dissociation–aggregation state of the Ag_29_(BDT)_12_(TPP)_4_ and Pt_1_Ag_28_(S-Adm)_18_(TPP)_4_ NCs. As shown in Fig. S7 and S8,† the ^31^P NMR spectrum of the Ag_29_(BDT)_12_(TPP)_4_ NC (without the addition of TPP) exhibits a broad peak (half-peak width ∼0.85 ppm), which narrows down continuously with the addition of more TPP (the peak width finally reduces to 0.17 ppm with a large excess of TPP in the Ag_29_(BDT)_12_(TPP)_4_ solution). The notable decrease in the peak width illustrates that the state of P is uniformalized; that is, the TPP dynamic dissociation–aggregation extent of the Ag_29_(BDT)_12_(TPP)_4_ NC in solution is remarkably suppressed with the addition of excess TPP. Furthermore, the maintained width of the ^31^P NMR peak (half-peak width by 0.02 ppm, shown in Fig. S9†) for the Pt_1_Ag_28_(S-Adm)_18_(TPP)_4_ with or without TPP addition demonstrates the TPP non-dissociated state of the Pt_1_Ag_28_(S-Adm)_18_(TPP)_4_ NC.

**Fig. 6:**
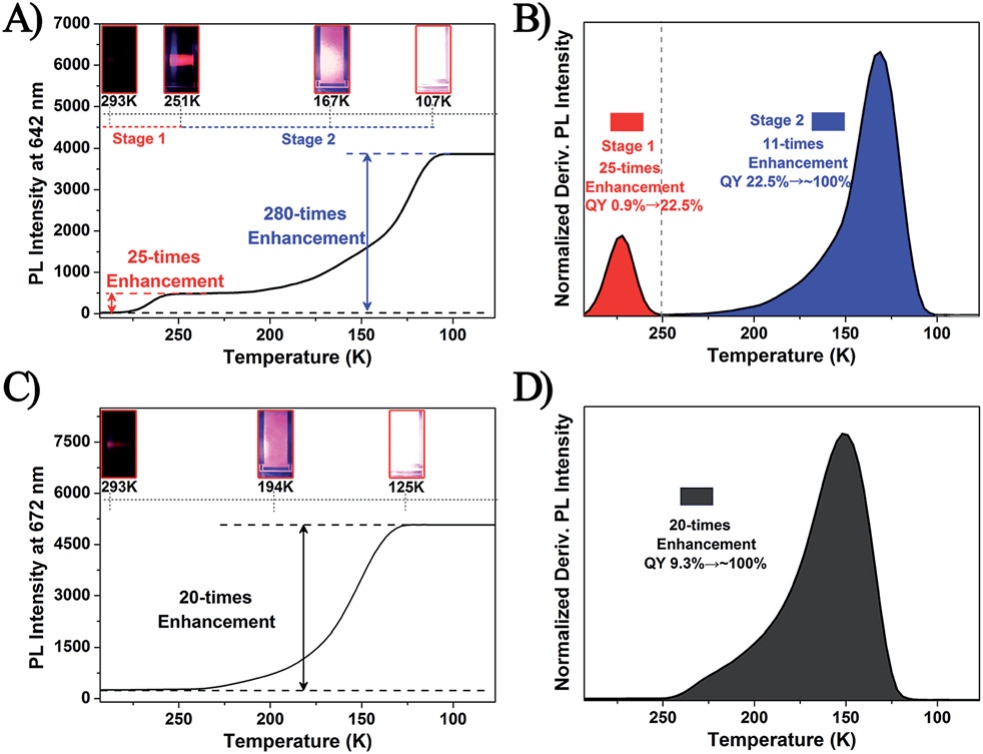
The PL intensity at the fixed points of (A) 642 nm for the Ag_29_(BDT)_12_(TPP)_4_ NC and (C) 672 nm for the Pt_1_Ag_28_(S-Adm)_18_(TPP)_4_ NC at different temperatures. Insets: the digital photographs of each NC in DMF solution under UV light. The derivative results for PL intensity of (B) Ag_29_(BDT)_12_(TPP)_4_ and (D) Pt_1_Ag_28_(S-Adm)_18_(TPP)_4_ NCs.

It is well known that the temperature would significantly influence the dissociation–aggregation dynamic equilibrium process. Thus, to further verify the above-mentioned AIE mechanism, the temperature–PL intensity correlation of the Ag_29_(BDT)_12_(TPP)_4_ and Pt_1_Ag_28_(S-Adm)_18_(TPP)_4_ NCs was monitored. As shown in Fig. 6A and B and S10,† the temperature-dependent fluorescence of the Ag_29_(BDT)_12_(TPP)_4_ NC shows two stages: (1) when the temperature is reduced from 293 K to 251 K, the fluorescence shows a 25-fold enhancement (in this state the UV-vis absorption is maintained, and thus the PL QY increases from 0.9% to 22.5%), and (2) the fluorescence intensity is increased significantly (a 280-fold boost comparing the 107 K data with the 293 K data) when the temperature is reduced to 107 K, and the UV-vis absorption presents a 2.5-fold enhancement (Fig. S11†). Accordingly, the PL QY increases almost to 100%. The fluorescence of the Ag_29_(BDT)_12_(TPP)_4_ NC appeared to be extremely bright at 107 K, which is in striking contrast to the nearly invisible fluorescence at room temperature (Fig. 6A, insets). In strong contrast, the non-dissociative Pt_1_Ag_28_(S-Adm)_18_(TPP)_4_ NC exhibits only one stage during the same temperature lowering process (Fig. 6C and D and S12†), and finally exhibits a 20-fold enhancement of PL intensity and 1.9-fold enhancement of UV-vis absorption (Fig. S13†). Thus, the PL QY of the Pt_1_Ag_28_(S-Adm)_18_(TPP)_4_ NC in the final stage is also nearly 100%. Furthermore, it should be noted that the single stage of Pt_1_Ag_28_(S-Adm)_18_(TPP)_4_ is similar to stage 2 of the Ag_29_(BDT)_12_(TPP)_4_ NC. Therefore, the PL boost in stage 2 of the Ag_29_(BDT)_12_(TPP)_4_ NC as well as in Pt_1_Ag_28_(S-Adm)_18_(TPP)_4_ should be completely induced by the suppression of thermal energy dissipation (or by the RIM pattern). Additionally, because the TPP dissociation process is much more sensitive to the temperature, the PL enhancement in stage 1 of the Ag_29_(BDT)_12_(TPP)_4_ NC should be mainly caused by the restriction of the TPP dissociation–aggregation process. Because this restriction process is easily influenced by the temperature variation, stage 1 is observed in the relatively high-temperature region (compared with stage 2 in the lower temperature region). To sum up, decreasing the temperature (from r.t. to 251 K) is effective in restricting the TPP dissociation–aggregation process which will lead to a significant enhancement in the fluorescence of the Ag_29_(BDT)_12_(TPP)_4_ NC in the solution state.

It should be noted that the PL enhancement of the Ag_29_(BDT)_12_(TPP)_4_ NC in the TPP addition process and temperature reduction process is different (13 times *versus* 25 times, with no increase in UV/vis absorption). In the TPP addition process, we find that the half-peak width (in the ^31^P NMR spectrum) of the Ag_29_(BDT)_12_(TPP)_4_ NC shows a continual decrease with the addition of TPP; however, the half-peak width in the final state is also wider than that in the Pt_1_Ag_28_(S-Adm)_18_(TPP)_4_ NC (0.17 *versus* 0.02 ppm). This suggests that the TPP dissociation–aggregation dynamic equilibrium in the Ag_29_(BDT)_12_(TPP)_4_ NC is not completely prohibited, but is just limited to a certain extent. In other words, the non-radiative pathways caused by the TPP dissociation–aggregation process still exist, even with the excess dose of TPP, which makes it difficult to reach the highest PL (with the non-dissociated state) of the Ag_29_(BDT)_12_(TPP)_4_ NC. By contrast, the non-dissociated state could be easily achieved in the temperature reduction process, and apparently, the Ag_29_(BDT)_12_(TPP)_4_ NC in the non-dissociated state (at 251 K) exhibits a higher PL QY than the final state in the TPP addition process (22.5% *versus* 11.7%).

## 4 Conclusions

In summary, a novel mechanism of aggregation-induced emission is discovered in nanoclusters, which involves the restriction of the ligand dissociation–aggregation process. The fluorescence intensity of Ag_29_(BDT)_12_(TPP)_4_ can be significantly enhanced (about 13-fold, quantum yield from 0.9% to 11.7%) *via* promoting the aggregation of TPP onto the easy-to-dissociate nanocluster surface. Furthermore, the TPP dissociation–aggregation dynamic equilibrium process of Ag_29_(BDT)_12_(TPP)_4_ is also restrained by reducing the temperature, which results in enhanced photoluminescence intensity (25-fold) in Ag_29_(BDT)_12_(TPP)_4_. In contrast, the same experiments performed on the non-dissociative Pt_1_Ag_28_(S-Adm)_18_(TPP)_4_ nanocluster do not show any PL enhancement. These different results are not caused by the presence of the Pt dopant, but by the different thiolate ligands (BDT *versus* S-Adm). The retained PL intensity in the case of Pt_1_Ag_28_(S-Adm)_18_(TPP)_4_ validates the aforementioned mechanism for the Ag_29_(BDT)_12_(TPP)_4_ nanocluster. Overall, this work presents a new mechanism of aggregation-induced emission in nanoclusters. In addition to previous studies on the enhancement of nanocluster photo-luminescence, this work will hopefully draw greater attention of optical and theoretical chemists to fully understand the photo-luminescence properties of metal nanoclusters. Future work will focus on extending this new AIE mechanism to other fluorescent nanoclusters.

## Conflicts of interest

There are no conflicts to declare.

## Supplementary Material

Supplementary information
