# Supplementary material for: Observation of a new type of aggregation-induced emission in nanoclusters
Source: Chem Sci. 2018 Feb 19;9(11):3062–8. doi: 10.1039/c7sc05317g (PMC5916020; doi:10.1039/c7sc05317g)
Supplement: Supplementary file 1 [file SC-009-C7SC05317G-s001.pdf]

Electronic Supplementary Material (ESI) for Chemical Science.
This journal is © The Royal Society of Chemistry 2018

Supporting Information

Observation of a new type of aggregation-induced emission in nanoclusters

Xi Kang,[a] Shuxin Wang,[a,*] Manzhou Zhu[a,*]

[a]Department of Chemistry and Center for Atomic Engineering of Advanced Materials, Anhui Province Key Laboratory of Chemistry for Inorganic/Organic Hybrid Functionalized Materials, Anhui University, Hefei, Anhui 230601, China.

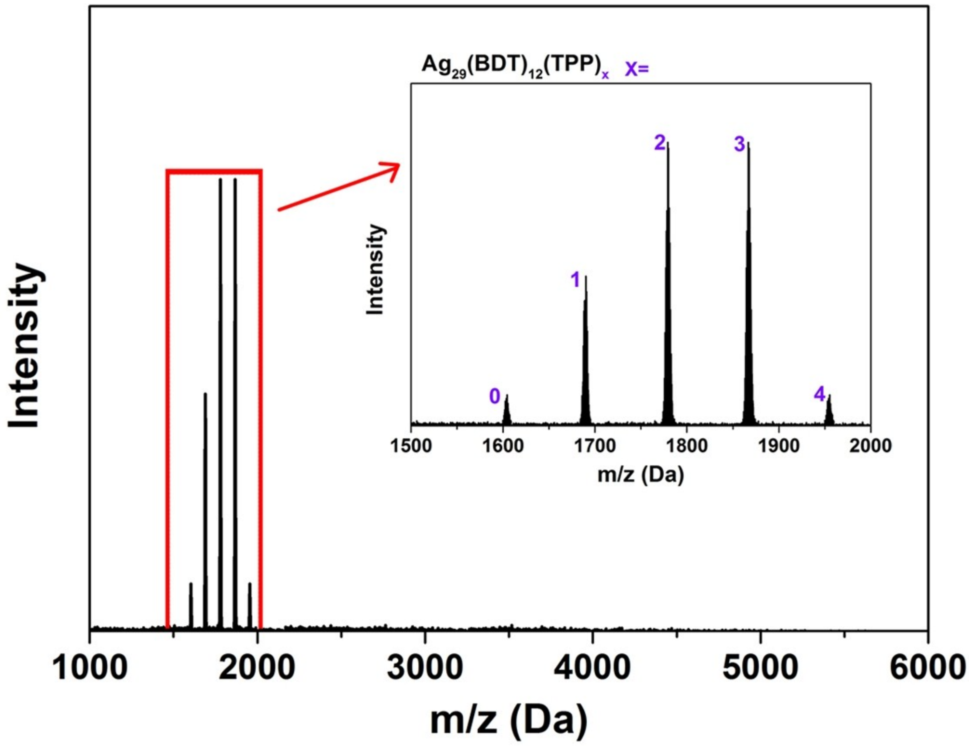

Fig. S1 Expansion of the ESI-MS spectrum in 1000-6000 Da (inset: 1500-2000 Da range) mass range of the $Ag_{29}(BDT)_{12}(TPP)_4$ nanocluster.

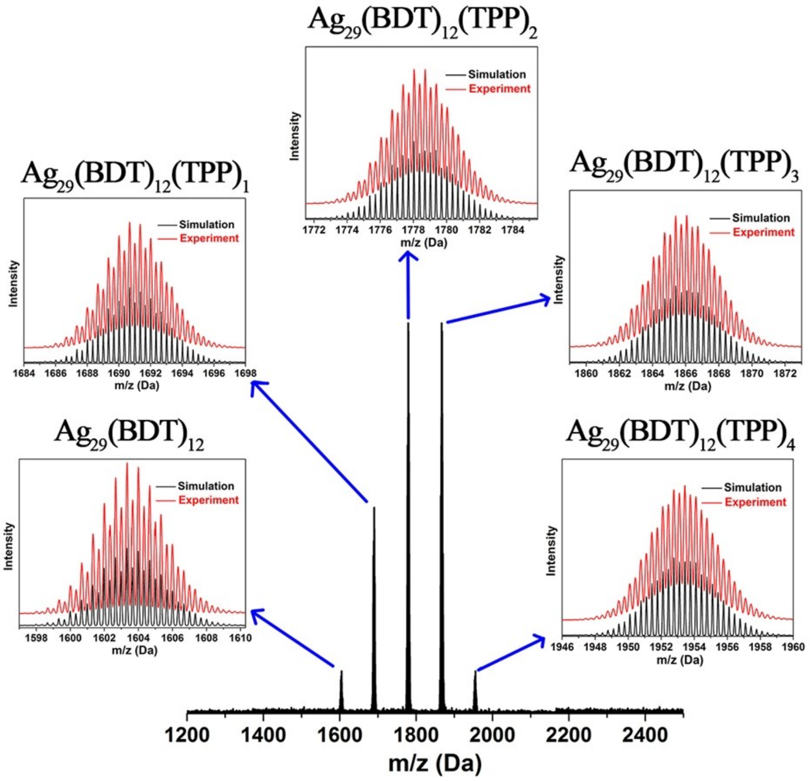

Fig. S2 ESI-MS spectrum in 1200-2500 Da mass range of the $Ag_{29}(BDT)_{12}(TPP)_4$ nanocluster. Insets: the experimental (red) and simulated (black) isotope patterns of $Ag_{29}(BDT)_{12}(TPP)_4$ nanocluster and its dissociated products.

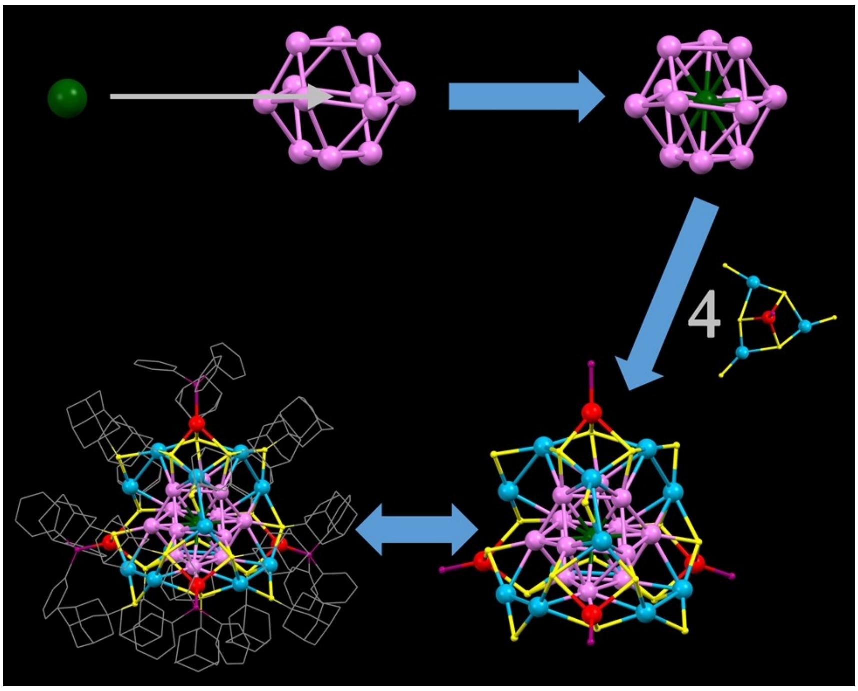

Fig. S3 Structural anatomy of $Pt_1Ag_{28}(S\text{-}Adm)_{18}(TPP)_4$ nanocluster. Color codes: green sphere, Pt; cerulean/red/violet sphere, Ag; yellow sphere, S; purple sphere, P; grey sphere, C.

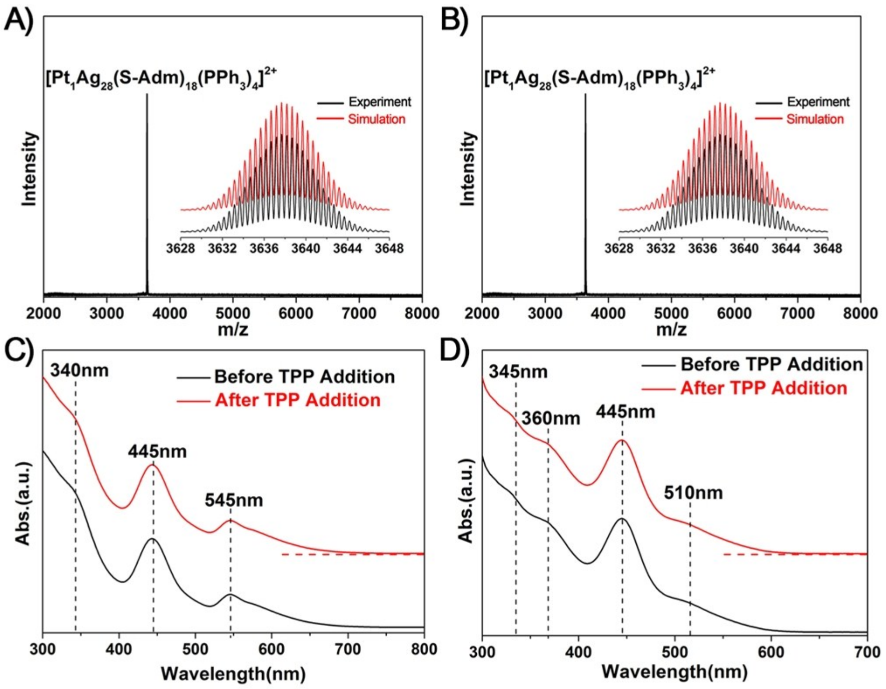

Fig. S4 ESI-MS results of $Pt_1Ag_{28}(S\text{-}Adm)_{18}(TPP)_4$ nanocluster A) before and B) after the addition of the TPP ligands. Unchanged UV-vis spectra of C) $Pt_1Ag_{28}(S\text{-}Adm)_{18}(TPP)_4$ and D) $Ag_{29}(BDT)_{12}(TPP)_4$ nanoclusters confirming the maintained structures and conformations of the nanoclusters.

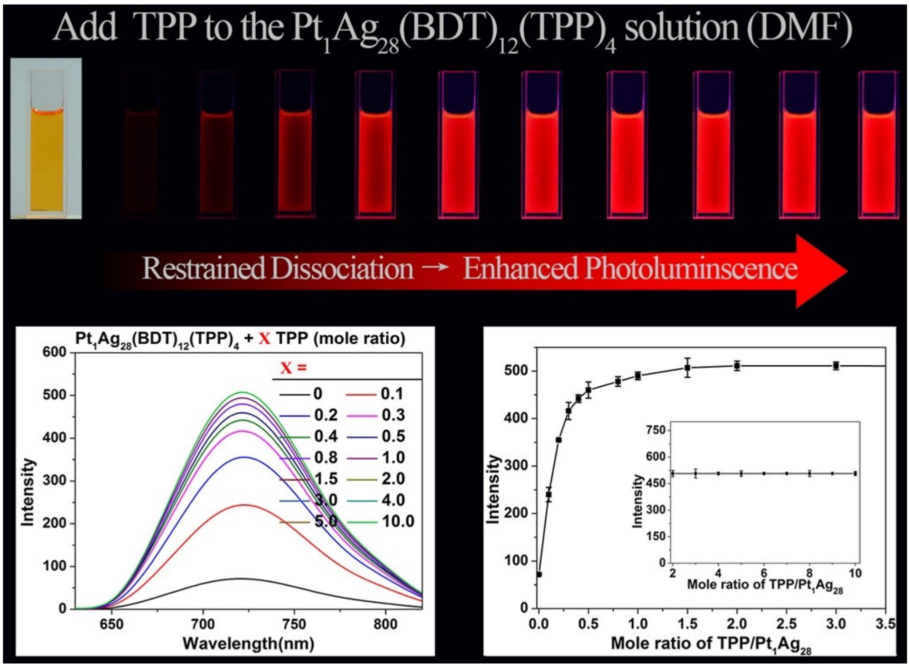

Fig. S5 PL variation trend of the $Pt_1Ag_{28}(BDT)_{12}(TPP)_4$ NC accompanied by the addition of different mole ratios of TPP. (Top) PL intensity variation monitored through the digital photographs of the $Pt_1Ag_{28}(BDT)_{12}(TPP)_4$ NC in solution state under UV light. (Bottom-left) PL intensity variation monitored by the fluorescence spectrometer. (Bottom-right) PL intensity variation monitored at the fixed-point of 720 nm.

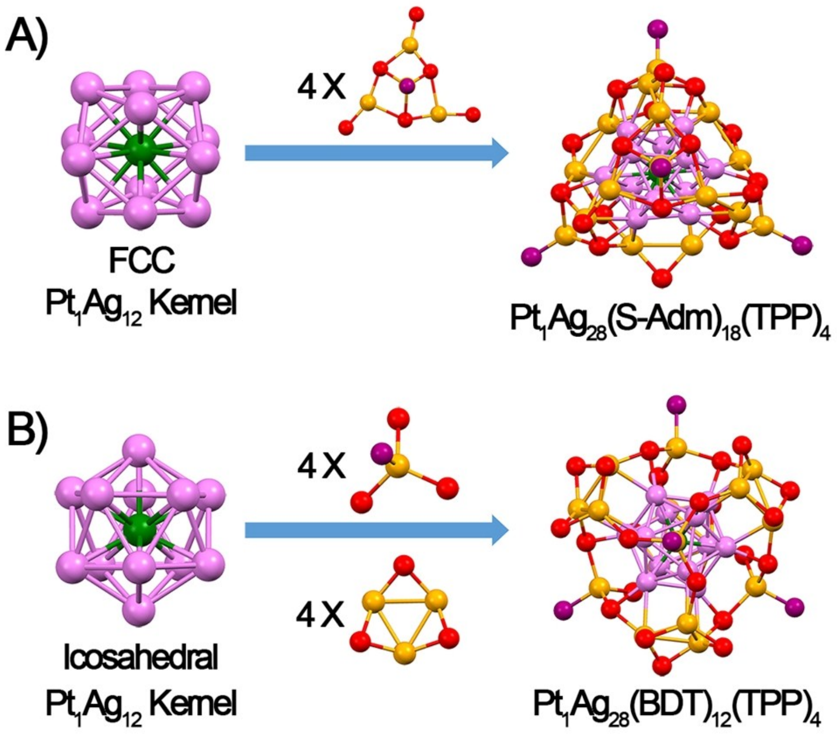

Fig. S6 Structural anatomies of the $Pt_1Ag_{28}(S\text{-}Adm)_{18}(TPP)_4$ as well as the $Pt_1Ag_{28}(BDT)_{12}(TPP)_4$ NCs. Color codes: green sphere, Pt; purple/orange sphere, Ag; red sphere, S; purple sphere, P. For clarify, the H and C atoms are not shown.

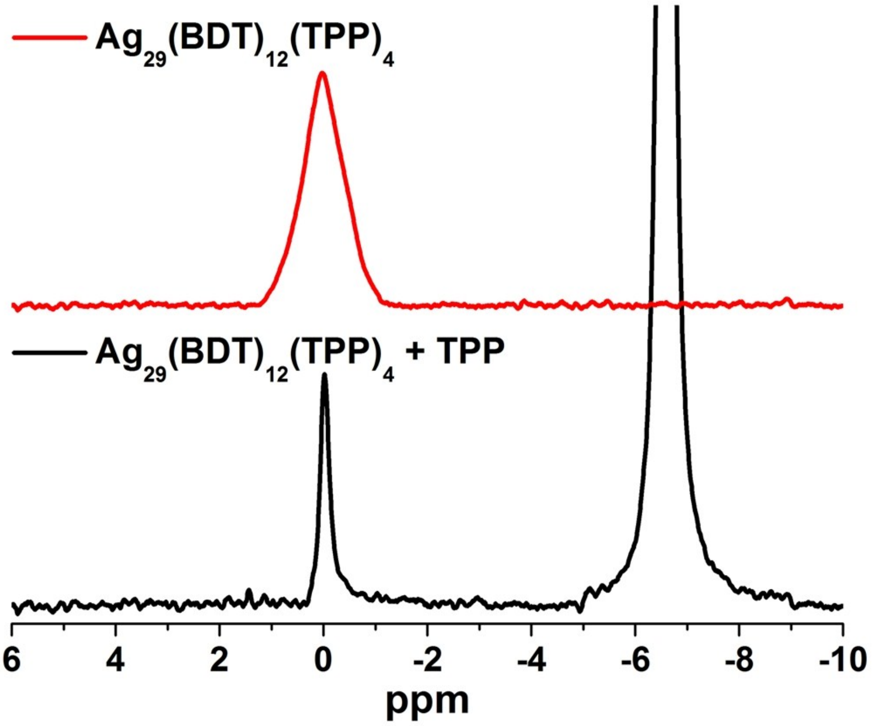

Fig. S7 ^{31}P NMR spectra (from 6 to -10 ppm) of pure $Ag_{29}(BDT)_{12}(TPP)_4$ nanocluster and the mixture of $Ag_{29}(BDT)_{12}(TPP)_4$ nanocluster and 2 mole ratio of TPP ligand.

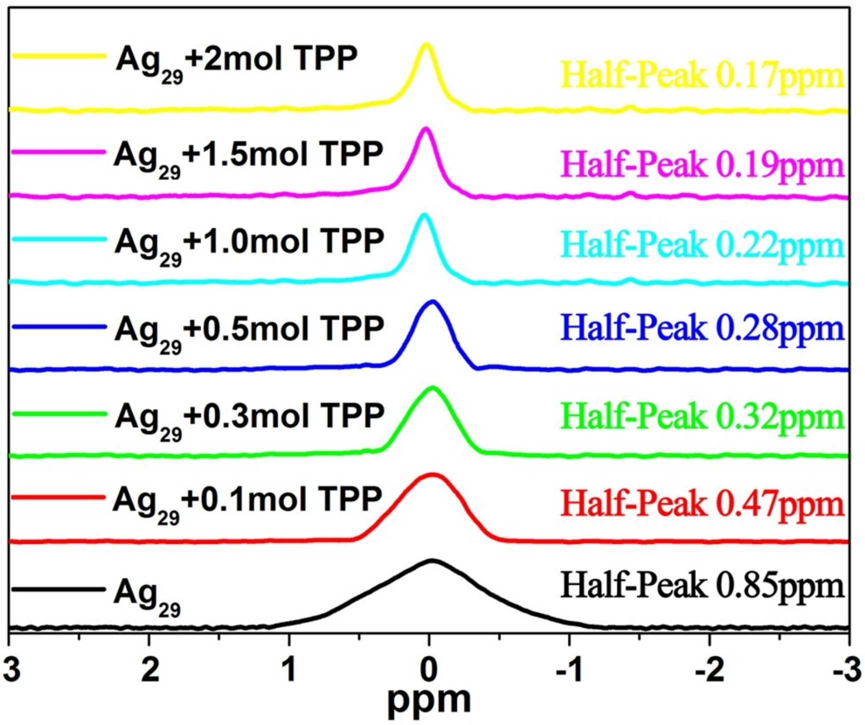

Fig. S8 ^{31}P NMR spectra (from 3 to -3 ppm) of pure $Ag_{29}(BDT)_{12}(TPP)_4$ nanocluster and the mixture of $Ag_{29}(BDT)_{12}(TPP)_4$ nanocluster and different mole ratio of TPP ligand.

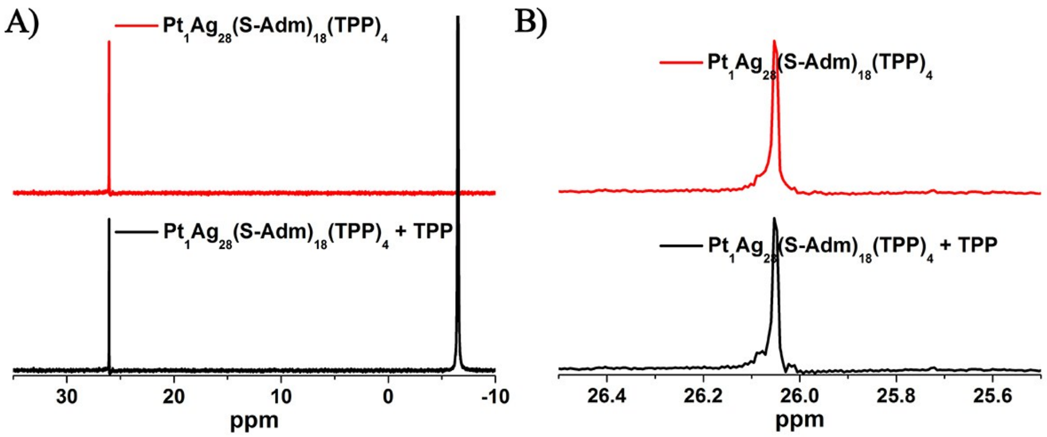

Fig. S9 ^{31}P NMR spectra (with different range, from 35 to -10 ppm or from 26.5 to 25.5 ppm) of pure $Pt_1Ag_{28}(S\text{-}Adm)_{18}(TPP)_4$ nanocluster and the mixture of $Pt_1Ag_{28}(S\text{-}Adm)_{18}(TPP)_4$ nanocluster and TPP ligand (2 mole ratio).

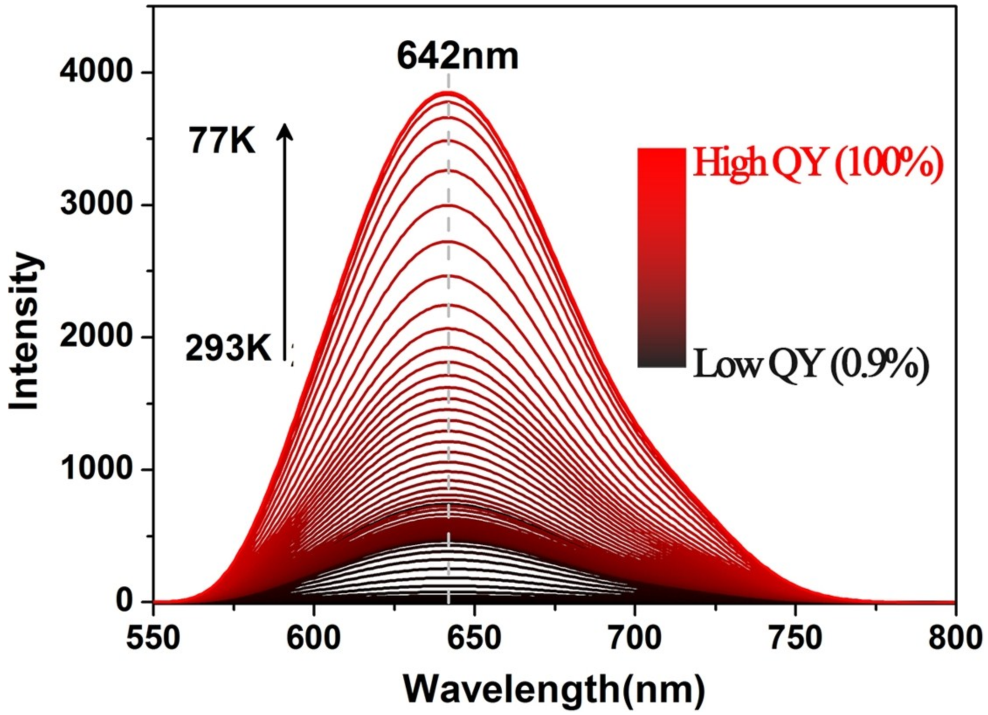

Fig. S10 Photoluminescence variation of $Ag_{29}(BDT)_{12}(TPP)_4$ nanocluster accompanying by the reduction of the temperature (from 293 K to 77 K, monitored per 3 K).

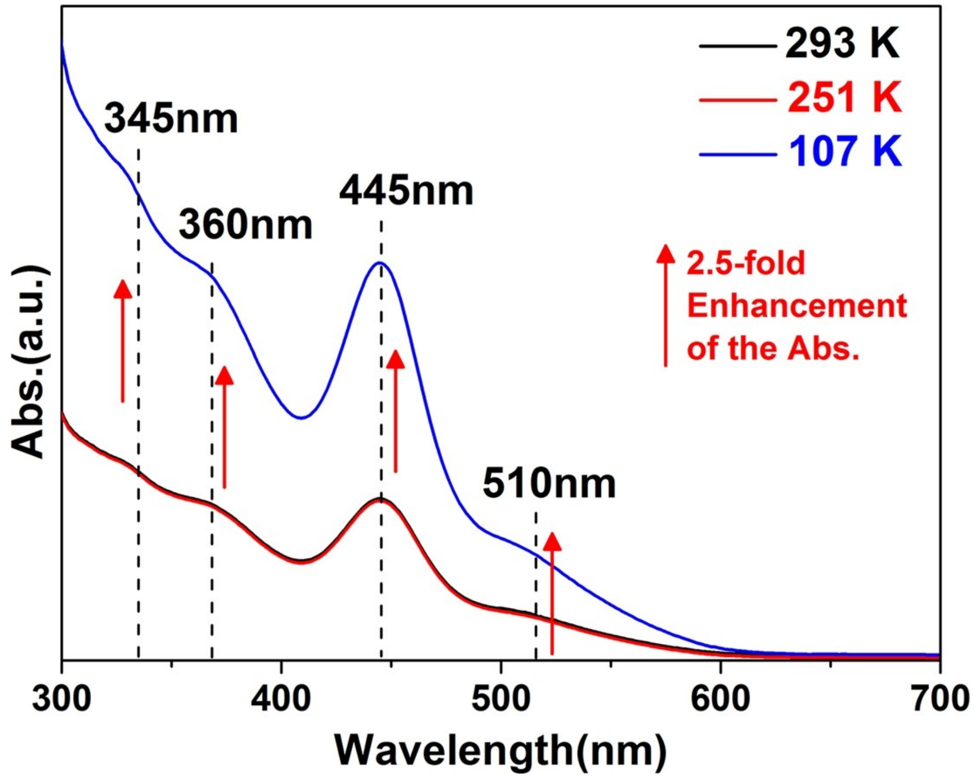

Fig. S11 UV-vis absorption variation of $Ag_{29}(BDT)_{12}(TPP)_4$ nanocluster accompanying by the reduction of the temperature (the "2.5-fold" is calculated from the absorption enhancement of each peak in the UV-vis spectra).

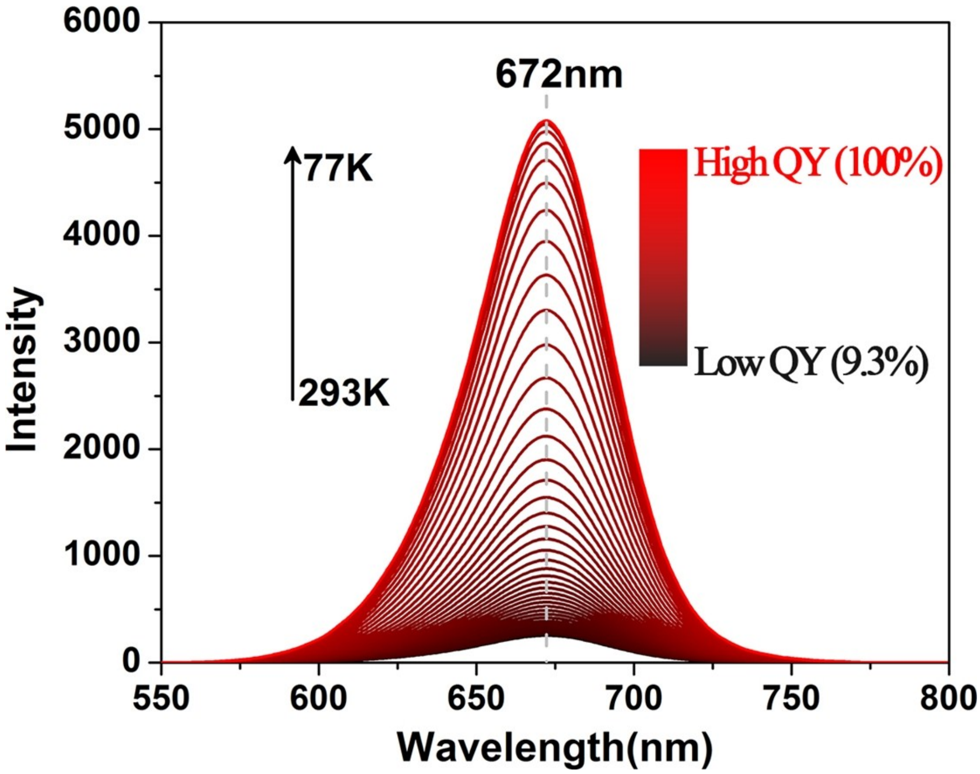

Fig. S12 Photoluminescence variation of $Pt_1Ag_{28}(S\text{-}Adm)_{18}(TPP)_4$ nanocluster accompanying by the reduction of the temperature (from 293 K to 77 K, monitored per 3 K).

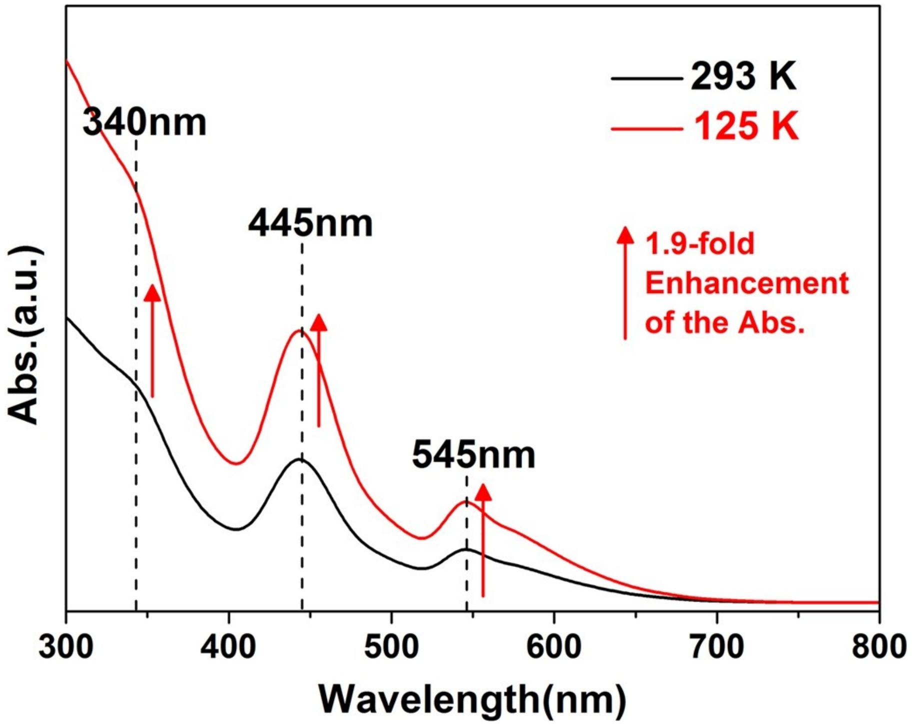

Fig. S13 UV-vis absorption variation of $Pt_1Ag_{28}(S\text{-}Adm)_{18}(TPP)_4$ nanocluster accompanying by the reduction of the temperature (the "1.9-fold" is calculated from the absorption enhancement of each peak in the UV-vis spectra).
